# Analyses of sex-based clinicopathological differences among patients with gastrointestinal neuroendocrine neoplasms in Europe

**DOI:** 10.1007/s00432-023-04711-4

**Published:** 2023-03-27

**Authors:** Henning Jann, Sarah Krieg, Andreas Krieg, Johannes Eschrich, Tom Luedde, Karel Kostev, Sven Loosen, Christoph Roderburg

**Affiliations:** 1 Department of Hepatology and Gastroenterology, Charité University Medicine Berlin, Augustenburger Platz 1, 13353 Berlin, Germany; 2 Clinic for Gastroenterology, Hepatology and Infectious Diseases, University Hospital Düsseldorf, Medical Faculty of Heinrich Heine University Düsseldorf, Moorenstrasse 5, 40225 Düsseldorf, Germany; 3 Center for Integrated Oncology Aachen-Bonn-Cologne-Düsseldorf (CIOABCD), Duesseldorf, Germany; 4 Department of Surgery (A), University Hospital Duesseldorf, Heinrich-Heine-University Düsseldorf, Moorenstrasse 5, 40225 Duesseldorf, Germany; 5 Epidemiology, IQVIA, Frankfurt, Germany

**Keywords:** Neuroendocrine neoplasia, Sex, Europe

## Abstract

**Background:**

Previous studies have found variations in cancer types, tumor progression, and disease outcomes between men and women. However, there is limited knowledge of the effect of sex on gastrointestinal neuroendocrine neoplasms (GI-NENs).

**Methods:**

We identified 1354 patients with GI-NEN from the IQVIA’s Oncology Dynamics database. Patients were derived from four European countries (Germany, France, the United Kingdom (UK), Spain). Clinical and tumor related characteristics including patients' age, tumor stage, tumor grading and differentiation, frequency and sites of metastases, as well as co-morbidities were analyzed as a function of patients´ sex.

**Results:**

Among the 1354 included patients, 626 were female and 728 were male. The median age was similar between both groups (w: 65.6 years, SD: 12.1 vs. *m*: 64.7 years; SD: 11.9; *p* = 0.452). UK was the country with the most patients, however, there was no differences in the sex ratio between the different countries. Among documented co-morbidities, asthma was more often diagnosed in women (7.7% vs. 3.7%), while COPD was more prevalent in men (12.1% vs. 5.8%). The ECOG performance states was comparable between females and males. Of note, the patients´ sex was not associated with tumor origin (e.g., pNET or siNET). Females were overrepresented among G1 tumors (22.4% vs. 16.8%), however, median proliferation rates according to Ki-67 were similar between both groups. In line, no differences in tumor stages was found and rates of metastases as well as the specific sites of metastases were similar between males and females. Finally, no differences in the applied tumor specific treatments between the both sexes became apparent.

**Conclusion:**

Females were overrepresented among G1 tumors. No further sex-specific differences became apparent, highlighting that sex-related factors might play a rather subordinate role in the pathophysiology of GI-NENs. Such data may help to better understand the specific epidemiology of GI-NEN.

## Introduction

Gender medicine focuses on the impact of sex on human physiology, pathophysiology, and clinical features of disease (Mauvais-Jarvis et al. 2020). Sex differences have been reported in different types of cancer, tumor aggressiveness, and disease prognosis (Haupt et al. 2021; Lopes-Ramos et al. 2020), but little is known about the effect of sex on gastrointestinal neuroendocrine neoplasms (GI-NENs).

GI-NENs are a diverse group of neoplasms that arise from the enterochromaffin cells of the gut (Lopes-Ramos et al. 2020). They are rare tumors, but their incidence has been increasing in recent years, which is mainly due to significant advanced in all fields of diagnostics for NEN (Ramesh et al. 2023). GI-NENs have several characteristics that distinguish them from other types of gastrointestinal tumors (such as pancreatic or colorectal carcinoma), including their slow growth, lack of symptoms for a very long period of time, and- in some cases- their high rate of hormone secretion. Despite their rarity, GI-NENs are clinically significant due to their significant impact they can have on a patient's general prognosis and quality of life. Recent studies have suggested that there are sex-specific differences in the incidence, presentation, response to treatment and prognosis of GI-NETs (Abdel-Rahman et al. 2022; Valent et al. 2021; Mogl et al. 2020).

Despite these concerns, no “sex-driven” diagnostic or therapeutic approaches are currently available. With the underlying goal to understand the impact of sex on epidemiology and clinical features of GI-NENs, we compared the general epidemiology and pathophysiological aspects of GI-NENs in four European countries (Germany, France, the United Kingdom (UK), and Spain) between male and female, using the IQVIA’s Oncology Dynamics database (Loosen et al. 2022a; Loosen et al. 2022b).

## Methods

### Database

For the purpose of this retrospective cross-sectional, we used the IQVIA’s Oncology Dynamics (OD) database, representing a cross-sectional partially retrospective survey collecting anonymized patient cases from a representative panel of oncologists (Zhao et al. 2012; Marchetti et al. 2017; Chambers et al. 2020)). The OD program collects anonymous patient-level data on treated cancer cases from multiple countries using a standardized online questionnaire. The questionnaire includes mandatory items and provides clear definitions and instructions to avoid errors and recall bias. Physicians are also asked to enter factual information from the patient’s medical records to avoid recall biases. To ensure input accuracy, the survey includes controlled code lists, multiple-choice questions, and interactive filters. Responses are immediately validated and checked for consistency, and any unexpected values prompt the participant to double-check their response. Physicians are asked to report recent cases they treated within the last week to prevent selective case submission. The program also performs additional validations and trend checks, and any anomalous values are corrected through discussion with the submitting participant (Alymova et al. 2022).

### Patient selection and study variables

This study looked at surveys of patients with neuroendocrine tumors (NET) in the small intestine, large intestine, stomach and gut (non-pancreatic) between January 1st, 2017 and March 31st, 2021 in four European countries: Germany, France, the United Kingdom, and Spain. The variables analyzed in the study were patients' age, co-morbidities, stage at diagnosis, site of metastases (liver, peritoneum, lung, bones), Ki-67 levels (< 2, 2–20, > 20) and ECOG performance status (0: asymptomatic, 1: symptomatic fully ambulatory, 2: symptomatic in bed less than 50%, 3: symptomatic in bed greater than 50% and 4: bedridden).

### Statistical analysis

The study compared the baseline characteristics of women and men using Chi-squared test for categorical variables and Wilcoxon test for age. Results were considered statistically significant if the p-value was less than 0.05. All analyses were done using SAS 9.4 software (SAS Institute, Cary, US).

## Results

### Baseline characteristics of study population

Overall, 1354 patients (626 women and 728 men) with neuroendocrine tumors of the gastrointestinal tract (GI-NET) from four different European countries were included. Baseline characteristics of the study population are given in Table 1. We found no significant differences of age between women (65.6; SD: 12.1 years) and men (64.7; SD: 11.9 years, *p* = 0.452). Most patients were aged 61–70 (33.9% of women and 34.6% of men) or 71–80 (26.7% of women and 24.5% of men). UK was the country with the most patients (37.1% of women and 41.6% of men), followed by Spain (22.8% of women and 19.2% of men), Germany (20.1% of women and 22.3% of men) and France (20.0% of women and 16.8% of men). Among documented co-morbidities, the prevalence of diabetes (18.2% of women and 15.1% of men), cardiac dysfunction (12.6 of women, 14.7% of men), atrial fibrillation (6.9% of women, 8.1% of men), and renal disease (5.6% of women, 4.8% of men) was similar between females and males. Interestingly, Asthma was found more often in females (7.7% women vs. 3.7% men, *p* = 0.002) while COPD was more prevalent in men (12.1% of men and 5.8% of women *p* < 0.001). The ECOG performance status did not significantly vary between males and females (*p* = 0.359), whereby the proportion of patients with ECOG 1 was 52.9% in women and 56.3% in men, followed by asymptomatic patients (ECOG 0; 30.6 of women and 30.1% of men), patients with ECOG 2 less than 50% of time (15.2% of women and 12.0% of men). Patients in bed greater than 50% of time (ECOG 3) or bedridden patients (ECOG 4) were very rare. The majority of women (85.9%) as well as men (86.9%) had inoperable tumor. The current line of therapy was in almost all cases the 1st line (79.1% of women and 81.6% of men). Regarding the most frequently used treatments, no clinically relevant differences between females and males were observed (Biologicals f: 67.2%, vs. *m*: 65.2%, *p* = 0.042; targeted therapies *f*: 18.7% vs. m: 16,8%, *p* = 0.032; classical chemotherapies f: 14,2% vs. *m*: 18,0%, *p* = 0.004).

**Table 1:** Baseline characteristics of study patients

Variable	Women	Men	*P* values
*N*	626	728	
Age (mean, SD)	65.0 (12.1)	64.7 (11.9)	0.452
Age group (*N*,%)			
≤40	19 (3.0)	24 (3.3)	0.757
41–50	62 (9.9)	64 (8.8)	
51–60	115 (18.4)	154 (21.2)	
61–70	212 (33.9)	252 (34.6)	
71–80	167 (26.7)	178 (24.5)	
> 80	51 (8.2)	56 (7.7)	
Country			
France	125 (20.0)	122 (16.8)	0.087
Germany	126 (20.1)	163 (22.3)	
Spain	143 (22.8)	140 (19.2)	
UK	232 (37.1)	303 (41.6)	
Co-Morbidities			
Diabetes	114 (18.2)	110 (15.1)	0.126
Renal disease	35 (5.6)	35 (4.8)	0.516
Atrial fibrilation	43 (6.9)	59 (8.1)	0.391
Cardiac dysfunction	79 (12.6)	107 (14.7)	0.268
Asthma	48 (7.7)	27 (3.7)	0.002
COPD	36 (5.8)	88 (12.1)	< 0.001
Venous thrombosis	5 (0.8)	7 (1.0)	0.750
Hepatitis C	9 (1.4)	15 (2.1)	0.387
ECOG performance status			
0-asymptomatic	192 (30.6)	219 (30.1)	0.359
1-symptomatic fully ambulatory	331 (52.9)	410 (56.3)	
2-symptomatic in bed less than 50%	95 (15.2)	87 (12.0)	
3-symptomatic in bed greater than 50%	8 (1.3)	11 (1.5)	
4-bedridden	0 (0.0)	1 (0.1)	
Current line of therapy			
1st line	495 (79.1)	594 (81.6)	0.731
2nd line	69 (11.0)	74 (10.2)	
3rd line	13 (2.1)	15 (2.1)	
4th line	3 (0.5)	3 (0.4)	
Adjuvant	37 (5.9)	35 (4.8)	
Neo-adjuvant	4 (0,6)	1 (0.1)	
Early stage/primary therapy	5 (0.8)	6 (0.8)	
Current resectability			
Inoperable	538 (85.9)	633 (86.9)	0.764
Operable	38 (6.1)	40 (5.5)	
Potentially operable	21 (3.4)	28 (3.9)	
Not known	29 (4.6)	27 (3.7)	

### Site of tumor origin and tumor differentiation

**Fig. 1:**
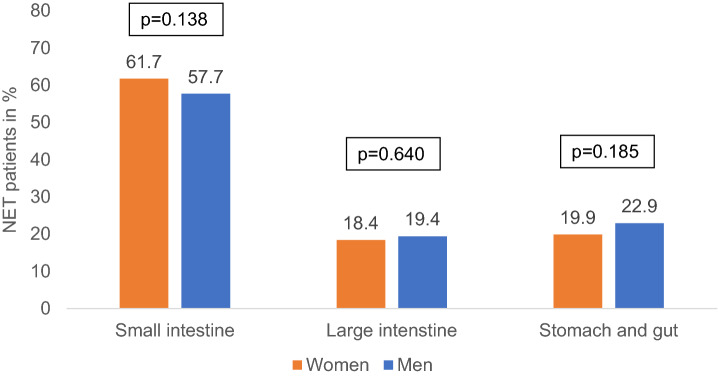
Cancer types of study patients by sex

Overall, the majority of patients had a tumor origin within the small intestine (women: 61.7%, men: 57.7%; *p* = 0.138, Fig. 1) followed by stomach and gut (19.9% of women, 22.9% of men, *p* = 0.185), and large intestine (18.4% of women, 19.4% of men, *p* = 0.640).

**Fig. 2:**
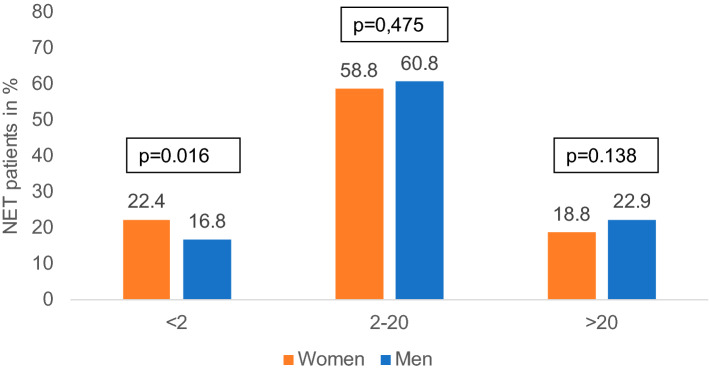
Marker of tumor proliferation, Ki-67 by sex (*n* = 1148)

Most patients in our analysis displayed a NET G2 (Ki-67 2–20%). However, there were important differences between women and men: women displayed a higher rate of well differentiated tumors compared to men (G1, Ki-67 < 2%: 22.4% vs. 16.8%, p = 0.016), since the proportion of highest rates of NET G3 (Ki-67 > 20%:) was slightly lower in women than in men (18.8% versus 22.9%) without statistical significance (*p* = 0.138, Fig. 2).

### Tumor stage (according to UICC) and pattern of metastases

**Fig. 3:**
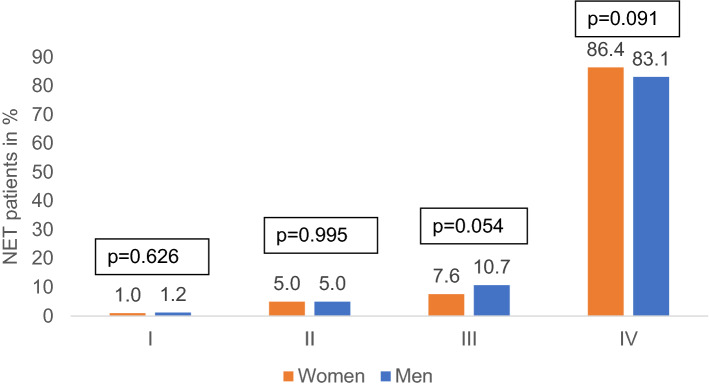
Cancer stage (according to UICC) at diagnosis by sex

**Fig. 4:**
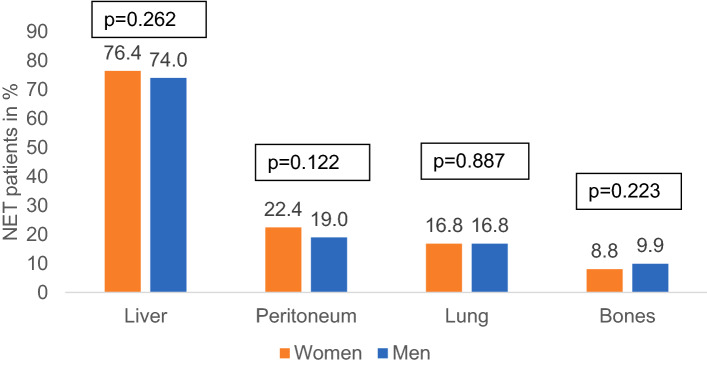
Site of metastasis by sex

In the whole population, almost all patients were diagnosed in a metastasized disease stage. When comparing women and men, no significant differences were observed in terms of stage IV (86.4% of women vs. 83.1% of men, *p* = 0.091). In other stages there were also no significant sex-related differences (Fig. 3). The most frequent sites of metastases documented were liver, peritoneum, lung, and bones. None of these sites of metastases was significantly affected by the patients’ sex (liver: 76.4% vs. 74.0%, *p* = 0.262; peritoneum: 22.4% vs. 19.0%, *p* = 0.122; lung: 16.8% vs. 16.8%, *p* = 0.887; bones: 8.8% vs. 9.9%, *p* = 0.223, Fig. 4).

## Discussion

In this cross sectional study, we analyzed clinicopathological features of GI-NENs with respect to the patients’ sex. Most importantly, we show that women demonstrate a lower Ki-67 index, while all other parameters including patients´ age, concomitant diseases, tumor origin, tumor stage, site of metastases, ECOG and applied treatment were similar between male and female, highlighting that—at least in our cohort of patients—the impact of the patients´ sex on both clinical and pathological features of NEN is limited.

Analyzing sex-specific differences in medicine is an emerging field that focuses on understanding the ways in which biological sex can impact the diagnosis, treatment, and outcomes of diseases such as cancer (Mauvais-Jarvis et al. 2020; Haupt et al. 2021; Lopes-Ramos et al. 2020), Research in this area has shown that there are important differences in the incidence, symptoms, and progression of various types of cancer between men and women (Mauvais-Jarvis et al. 2020; Haupt et al. 2021; Lopes-Ramos et al. 2020). Additionally, sex-specific hormones such as estrogen and testosterone can play a role in the development and progression of certain types of cancer (Rubin et al. 2020; Roshan et al. 2016). As a result, sex-specific approaches to cancer diagnosis and treatment are being developed and studied, with the goal of improving outcomes for both men and women. This includes personalized medicine approach where patients are treated based on their individual characteristics, rather than applying a one-size-fits-all approach. By understanding these differences, medical professionals can tailor treatments to best suit the needs of each patient, leading to better outcomes and improved quality of life for those with cancer.

Specifically regarding NEN, the available studies on sex-specific differences are limited, and available data are at least partially inconsistent. In the US and Canada, pNENs are more common in men, while in Italy they tend to occur more often in women. This raises questions about potential differences in prevalence based on sex or geographical region and suggests that environmental factors may play a certain role in this context (Fu et al. 2022). We demonstrate that the site of origin (e.g., pNET vs. siNET) is similar in male and female. Interestingly, a recent analysis on NEN patients from China suggested that the age at diagnosis was younger in females compared to males, which is not supported by our own analyses, while other reports on this question reported controversial data (Blažević et al. 2022; Muscogiuri et al. 2020), highlighting the large differences in epidemiological analyses on patients with NEN. In our analyses we demonstrate that female patients are diagnosed at higher rates with G1 (Ki-67 < 2%) tumors than male patients, which is strikingly supported by similar data from an analysis on pNET patients (Muscogiuri et al. 2020). Notably, the significance of this finding is questioned by the fact that the median Ki-67 was similar between both sexes. The latter is in line with a recent analyses including 559 siNET patients showing that there were no statistically significant differences between male and female patients in tumor grade or serum chromogranin A (CgA) level (Blažević et al. 2022) as well as other analyses from Italy (Muscogiuri et al. 2020) and China (Fu et al. 2022). Recently, it was found that females have a higher rate of insulinomas (Patel et al. 2013; Service and F.J., 1991), which may cause them to experience symptoms and be diagnosed earlier, potentially leading to an earlier stage diagnosis. However, neither our analysis nor previous analyses (Fu et al. 2022; Blažević et al. 2022) revealed significant differences in tumor stages or sites of distant metastases between males and females. Finally, we analyzed differences in co-morbidities of male or female GI-NEN patients. We found higher rates of asthma but lower rates of COPD in females. This observation is interesting in the context of a previously reported association between tobacco smoke and pNENs (Vigneri et al. 2016; Capurso et al. 2009; Halfdanarson et al. 2014; Zhan et al. 2013). However, these studies did not look into whether men and women have different levels of susceptibility to tobacco smoke in relation to pNENs and the clinical effect and/or relevance is not understood to date. Previous reports also reported rather inconsistent pictures and suggested differences in cardiovascular diseases (Muscogiuri et al. 2020). Thus, in the light of our report and the lack of consistent data from other analyses, overall, the sex-specific differences in clinicopathological features or response to treatment (Mogl et al. 2020) of GI-NEN appear to be limited or are at least not sufficiently understood.

In a recent study we have described variations in both clinical and pathological characteristics of patients with GI-NENs among European countries (Ramesh et al. 2023). Here, we specifically analyzed the impact of the patients´ sex on these factors. These data integrate very well with the current discussion of sex-related factors in cancer patients. Among the total of 1354 patients (626 women and 728 men) that were part of our present analysis, 289 (21.3%) were derived from Germany, 247 (18.2%) from France, 283 (20.9%) from Spain and 535 (39.5%) from the U.K. As described in the introduction section of this manuscript, GI-NEN belong to the rare diseases with an annual incidence of 0.48/100000 (Dasari et al. 2017; Lahner et al. 2022). In this context, we were able to examine a relatively large cohort of patients derived from the IQVIA’s Oncology Dynamics database, representing an important strength of this analysis. Nevertheless, we recognize important limitations of our study that clearly need to be discussed: Most importantly, many information is lacking. As an example, data on a potential hereditary background as well as data on the functional activity are not available within the Oncology Dynamics database. Moreover, due to recent changes in the WHO classification of NEN, at least some patients with NEC might have been misclassified and rather represent NET G3, it is therefore likely that the database might not be representative for the whole spectrum of GI-NENs. Moreover, it is important to note that the database only included patients who were treated with drugs and the questionnaire used was not specifically designed for the research being conducted. The database also has limitations such as missing information on genetic factors and socioeconomic status. Additionally, it should be kept in mind that this study can only show associations and not causality, and it does not compare to other established databases. Finally, there is a possibility that different presentation patterns may lead to the observed sex-based differences, and the limited availability of covariates documented in the study hinders the ability to control for confounding factors. Despite these limitations, the database has been used in many studies and has been found to be suitable for clinical research (Loosen et al. 2022a).

In summary, our data highlight that GI-NENs demonstrate a higher proportion of females among low proliferative patients. In contrast to previous analyses, all other parameters including patients´ age, concomitant diseases, tumor origin, tumor stage, site of metastases, ECOG performance status and applied treatment were equally distributed between both sexes. The results presented here are important for getting a more complete picture on the epidemiology of GI-NENs and might trigger further epidemiological studies ultimately leading to a better clinical management of patients with NENs.

## Data Availability

The datasets generated during and/or analysed during the current study are available from the corresponding author on reasonable request.

## Abbreviations

GI-NENs: Gastrointestinal neuroendocrine neoplasms
NEN: Neuroendocrine neoplasms
OD: Oncology dynamics database
NET: Neuroendocrine tumors
ECOG: Eastern cooperative oncology group
GI-NET: Gastrointestinal neuroendocrine tumors
UK: United Kingdom
COPD: Chronic obstructive pulmonary disease
G: Grade
UICC: Union International Contre le Cancer
US: United States
pNET: Pancreatic neuroendocrine tumors
siNET: Small intestine neuroendocrine tumors
CgA: Chromogranin A
WHO: World Health Organization
NEC: Neuroendocrine carcinoma
